# Women’s orgasm obstacles: A qualitative study

**Published:** 2017-08

**Authors:** Maryam Nekoolaltak, Zohreh Keshavarz, Masoumeh Simbar, Ali Mohammad Nazari, Ahmad Reza Baghestani

**Affiliations:** 1 *Student Research Office, Depatment of Midwifery and Reproductive Health, School of Nursing and Midwifery, Shahid Beheshti University of Medical Sciences, Tehran, Iran.*; 2 *Depatment of Midwifery and Reproductive Health, School of Nursing and Midwifery, Shahid Beheshti University of Medical Sciences, Tehran, Iran.*; 3 *Midwifery and Reproductive Health Research Center, Shahid Beheshti University of Medical Science, Tehran, Iran.*; 4 *Department of Nursing and Midwifery, Shahrood University of Medical Science, Shahrood, Iran.*; 5 *Department of Biostatistics, School of Allied Medical Science, Shahid Beheshti University of Medical Sciences, Tehran, Iran.*

**Keywords:** Female, Orgasm, Obstacles, Sexual satisfaction, Qualitative study

## Abstract

**Background:**

Woman’s orgasm plays a vital role in sexual compatibility and marital satisfaction. Orgasm in women is a learnable phenomenon that is influenced by several factors.

**Objective:**

The aim of this study is exploring obstacles to orgasm in Iranian married women.

**Materials and Methods:**

This qualitative study with directed content analysis approach was conducted in 2015-2016, on 20 Iranian married women who were individually interviewed at two medical clinics in Tehran, Iran.

**Results:**

Orgasm obstacles were explored in one category, 4 subcategories, and 25 codes. The main category was “Multidimensionality of women’s orgasm obstacles”. Subcategories and some codes included: Physical obstacles (wife’s or husband’s boredom, vaginal infection, insufficient vaginal lubrication), psychological obstacles (lack of sexual knowledge, shame, lack of concentration on sex due to household and children problems), relational obstacles (husband’s hurry, having a dispute and annoyance with spouse) and contextual obstacles (Irregular sleep hours, lack of privacy and inability to separate children’s bedroom from their parents, lack of peace at home).

**Conclusion:**

For prevention or treatment of female orgasm disorders, attention to physical factors is not enough. Obtaining a comprehensive history about physical, psychological, relational and contextual dimensions of woman’s life is necessary.

## Introduction

Orgasm is a temporary peak of pleasant sexual sensation that is associated with some physiological changes in the body. Orgasm in men typically accompanies ejaculation that makes this phenomenon more recognizable. However, reaching the orgasm in women is not as easy as in men and for some women, it is difficult to know if orgasm has occurred or not (1). There are different approaches in dealing with sexual issues; one of which is “Biopsychosocial” approach presented by Rossi in 1994. The foundation of this approach is the interactions and influences of the three biological, psychological and social dimensions on human sexual affairs. In this framework, it is possible to classify those factors that can affect orgasm (2). 

Biological factors, including variations in physiology and neuroanatomy of the clitoris and anterior vaginal wall, thickness of urethrovaginal space and the distance between clitoris and urethral meatus may justify why some women reach orgasm more easily and some others with more difficulty. Also, Drugs, particularly selective serotonin reuptake inhibitors (SSRIs), are associated with a delay in orgasm (3-6). Psychological and relational factors such as introversion, emotional instability, lack of openness to new experiences, feeling guilty for being joyous, sexual myths, anxiety and depression and anti-masculine feelings can affect female orgasm (7-10).

Relational factors and inability to talk about sexual activities with a spouse can be observed in women with orgasm problems (11, 12). Similarly, the degree of reproach and less receptivity has been reported more frequently in marital relationships of women with orgasm disorder (13). Instead, those couples with a larger number of sexual relationships and more diverse sexual activities together will experience higher sexual satisfaction and a better orgasm (14). Social concepts, sociocultural factors and the existing sexual myths in a society can have an impact on female orgasm (8,15, 16). Woman’s orgasm impacts on her sexual compatibility with the spouse, sexual satisfaction and happiness in marital life (17-20).

In a qualitative study by khoei and colleagues, orgasm components from Iranian women's views were explored and the concept of “Romantic Love“ was considered as the main interpretation of sexual pleasure by Iranian women (21). Qualitative researches on orgasm among Iranian women are necessary and important for the following reasons: Woman’s orgasm is a subjective experience; each woman experiences orgasm differently and she may experience a variety of orgasms over her course of life, therefore a qualitative research is an appropriate method to identify this unique experience (22). On the other hand, a large number of Iranian women treat sexual issues with silence due to shame and sexual taboos. Safe and confident conditions of an in-depth qualitative research can help these women to speak of their untold sexual issues. 

Moreover, there are several subcultures in Iran. As cultural context affects sexual issues, including female orgasm, culture-based qualitative research in this field seems necessary. Besides, by the growth of social media, the fact of orgasm has been obscured for people and therapists in an aura of exaggeration by porn movies and it is essential to make clarifications in this respect through qualitative researches. Therefore, in order to prevent and treat orgasm disorders, it is necessary to analyze female orgasm obstacles in her real life by performing qualitative researches.

This qualitative research aims to explore the factors affecting Iranian women's orgasm and to identify obstacles to female orgasm. It is hoped that the results of the current research will improve women's orgasm and promote their sexual satisfaction and family stability.

## Materials and methods

During the last 10 years, family researchers have conducted qualitative studies in addition to quantitative studies. Emphasizing the complexity of the issues, instead of analyzing numbers, they have focused on rich descriptions. Also based on pluralism, combining qualitative and quantitative research (Mixed method study) is increasing (23). This qualitative research is part of a mixed method study. In the first phase, the participants stated that reaching orgasm in sexual relationship greatly affects their sexual satisfaction and compatibility. Therefore, in the next step, the factors influencing orgasm were considered by the researchers. 

**Procedure**

The researcher had obligation to observe all ethical principles such as secrecy, anonymity, and allowing the participants to leave the study at any desired time. Research setting included two medical clinics in Tehran. Purposive sampling was started and continued until data saturation occurred (24). All the interviews were performed by the same person (the first author). Duration of the interviews varied from 20-90 min, depending on the participants’ interest. Semi-structured interviews were conducted with guiding questions (25). The main guiding questions were as follows: How is your sexual relationship with your spouse? In your sexual relationships, what factors affect reaching orgasm?

**Participants**

The participants in this study included 20 married women from different ages, occupations, educational level, years of marriage, and numbers of children. These women were referred to medical clinics and had been visited by a general practitioner for non-sexual reasons and then, they were invited and directed to participate in this research. 

The Inclusion criteria were: being female and married, living in Tehran, having at least one year of marital life with a spouse, having fluency in the Persian language, being interested in participation and being able to establish a relationship and express her sexual life experiences. The exclusion criteria included: individual’s self-report on her physical or mental disease or using drugs that would affect her sexual performance. The youngest participant was 19 years old and the oldest was 48. Their duration of marriage varied from 1-30 yr. The number of children was between 0 and 3. The lowest educational level was illiterate and the highest was a Ph.D. degree. Table I shows the socio-demographic characteristics of the participants.

**Ethical consideration**

The ethical code is SBMU2.REC.1394.73. Verbal or written informed consent was taken from all the participants.

**Data analyzing**

The interviews were recorded and transcribed verbatim. First, the audio interviews were typed carefully and imported into the MAXQDA-10 software. The participants’ sentences formed the meaning units. Data analysis began with frequent reading and a general sense perception, continued with codes extraction and putting them in the subcategories and ended in the derivation of the main category. In order to name the subcategories, a literature review was used. 

According to prior researches on orgasm and within the framework of biopsychosocial theory, the factors affecting sexual affairs including orgasm were divided into physical, psychological and contextual factors. Then the codes extracted from the meaning units were placed in the subcategories derived from literature review. This method is called directed content analysis that is applied when previous theories or research on a phenomenon are not complete and comprehensive and it needs further details (27). In this study, prior theories or studies on female orgasm in Iranian culture were not thorough and further description was necessary. 

Guba and Lincoln criteria, including credibility, transferability, confirmability and dependability were used (28, 29). Member-checking and peer- checking were done to ensure the credibility of the research data. For member-checking, during the interview, the participant’s words were fed-back to herself to confirm them. On the other hand, 2 participants checked the transcripts and emerging codes from the interviews. In peer-checking, coding and categorizing process was checked by the other members of the research team and discussion about disagreement parts continued until agreement was obtained. Also, long-term engagement, adequate time allocation and proper communication with participants were carried out.

In order to reach transferability, detailed and thick descriptions about the environment, participants and their non-verbal behaviors were written by the researcher. Also, demographic information of the participants was reported and sampling with maximum variation was done to increase the transferability. Confirmability was established through external checking and verifying the coding and categorization process by two experts in the field of qualitative research and sexual health. In order to evaluate the consistency and dependability, study process was explained in detail and research memo was written exactly.

## Results

In this study, 20 married women from different ages, occupations, educational levels, years of marriage and numbers of children, spoke about the factors obstruct their orgasm. Following the content analysis, the obstructive factors of female orgasm among Iranian woman were developed in 1 category, 4 subcategories, and 23 codes. Tables II demonstrates the category, subcategories, and codes obtained in this study. In this study, 8 women had a history of lack of orgasm: one woman (Participant 10) had never experienced orgasm in sexual relationship with her husband, 3 women (Participant 5, Participant 20, Participant 13) had secondary orgasmic disorder and 4 women (Participant 1, Participant 4, Participant 7, and Participant 14) had improved primary orgasmic disorder.

**Obstacles to women’s orgasm**

In response to this question" In your sexual relationships, what factors affect reaching orgasm?” participants mentioned facilitator factors and obstructer factors. This article is about factors that play as obstacles to women’s orgasm. These obstacles can be classified in 4 subcategories of physical, psychological (individual and relational) and contextual factors.

**Physical obstacles**

**Physical fatigue of the husband or wife**

Participant 9 stated, “if my husband was less tired and could rest more, our relationship was more satisfactory and I could reach orgasm more easily”. Participant 2 also said “taking care of my 2-month and 3-year old babies make me really tired, fatigue has affected the frequency of our sexual relationships and the degree of our pleasure”.

**Vaginal infection**

Participant 16 stated, “whenever I have a vaginal infection, I cannot reach orgasm”.

**Insufficient vagina** **l lubrication**

Participant 5 said: "Six months after my delivery, we still have not made a sexual relationship. After giving birth, I don’t have vaginal lubrication and I have no desire for sex."

**Psychological obstacles**

**Lack of sexual knowledge**

Participant 1 expressed a lack of sexual knowledge as the main cause of not reaching orgasm during the first 6 months of her marriage: “I think the cause of my failure to reach an orgasm, was insufficient sexual knowledge and being inexperienced”. Participant 7 said: “After one year of marriage, we found an audio file from a doctor, who explained about sexual relationships, after hearing it and understanding how to make a sexual relationship and its arousing acts, we achieved sexual pleasure. Maybe if we had prior knowledge or we've seen a training movie about sex processes, we could understand the joy of sex sooner.

**Shame**

Shame and failure to express her sexual demands have been an obstacle to orgasm for Participant 18: “It was 10 years after our marriage that I finally put away the shame and talked about some of my sexual demands with my husband. Since then, I have enjoyed my sex with my husband”. Participant 8 also said: “After 4 years of marriage, when I had a child, I could talk about my sexual needs more easily, and then we reached a desirable sexual compatibility. Before that, my husband tried, but I did not have complete satisfaction. If I was not shy and I spoke earlier, I had better conditions". Participant 14 had been embarrassed to perform some sexual behaviors: "Early in the marriage, I was ashamed of doing some sexual behaviors, but now I am not because I have a more intimate relationship with my husband".

**Lack of concentration**

- Concerns about the household and children problems.

Participant 19, Participant 16 and Participant 20 mentioned the lack of concentration as an obstacle to reaching orgasm: Participant 19 stated that “It rarely happens that I don’t reach the climax and that is when I have been involved in everyday activities.” In this regard, Participant 16 said, “Thinking about my child’s academic problems make me uninterested in sex and orgasm.” Participant 20 said, “When I am mentally involved, for example, when I’m worried about the economic condition of our household or some problems that my child are facing, I don’t feel relaxed and I’m not in the mood for sex and if I have an intercourse, I will not enjoy it.”

- Concerns about children’s imagination about parent's sex

Participant 10 and Participant 20 minimized their sex with their spouse due to fear of their children’s imagination and do not reach orgasm most of the times. Participant 10 said: “Now at home, we have 2 bedrooms, but just like before, we sleep next to the children in the living room, the kids have grown up and they may think that why our parents are not sleeping next to us anymore? Why do they go to a private bedroom? What do they do?!.However, I have never enjoyed my sexual life.” Participant 20 also said: “We have a private bedroom and we sleep there, but we do not close the door of the bedroom, because my son is 22 years old and I fear that he will be promiscuous, he may think what my parents are doing?

- Wife’s concerns about her husband’s satisfaction

Worrying about her husband’s satisfaction has prevented Participant 14’s from having pleasure: “During the first year of our marriage, I hardly reached the climax because I frequently was thinking if my husband is satisfied with me or not”.

**Fear of sexual intercourse at t** **he beginning of marriage**

Fear of sexual intercourse during the early days of marriage was an obstacle for Participant 1, Participant 12 and Participant 4 to reach an orgasm. Participant 1 said “in the early days of marriage, I was afraid of sex. I was afraid that my hymen was going to be deflowered and it could be painful”. Participant 12 also stated that “At the beginning of the marriage, I was really afraid of sex and I did not enjoy it”. Participant 4 has seen a sexy movie in her adolescence and was afraid of sex. This fear was problematic for her during the early years of her marriage: "When I was in high school, one day, in my friend's house, I saw a sexy movie. When I saw that scene, I was really afraid of marriage and sex.”

**Fear of pregnancy**

For Participant 3, fear of pregnancy during the first year of marriage was a major obstacle that did not let her enjoy her sexual relationship: “Over the first year of marriage, we had a withdrawal method of contraception and many times we left the relationship unfinished due to fear of getting pregnant so that I could not experience the climax”.

**Anxiety of orgasm failure recurrence**

Participant 1, who did not experience orgasm during the first six months of marriage and was able to reach the climax after referring to a consultant, stated: “During the early days of therapy, I was afraid that I will not reach an orgasm again, however, with focusing on positive thoughts I could overcome this anxiety and gradually our pleasurable relationships grew more and more.”

**Relational obstacles**

**Husband’s hurry**

Husband’s hurry has been an important obstacle to orgasm for Participant 13: “It would be better if I did not respond to his every need from the very beginning. It would be better if I made him take a shower, put on perfume and then make love, but I satisfied his needs immediately and he became cold when I just started to feel my needs. Definitely, our relationship had problems that made me so disillusioned. My husband does not know a woman’s need and how to caress. He just wants to establish a quick intercourse.”

**Having a dispute and annoyance with spouse**

Quarrel and annoyance at the spouse did not let Participant 17, Participant 20 and Participant 15 perceive their sexual pleasure. Participant 17 said, “If I have emotional problems or conflicts with my husband, I do not reach the climax.” Participant 20 also expressed “If I have a dispute or quarrel with my husband during the day, my mind will be engaged and I cannot reach an orgasm at night.” Participant 15 also said, “Quarrel and annoyance at my husband will mitigate my sexual pleasure.” While dispute with a spouse was an obstacle for Participant 17, Participant 20 and Participant 15 to perceive orgasm, Participant 16 totally would experience orgasm easily and if she was annoyed with her spouse, she did not avoid sex with her husband and even reached orgasm in the case of annoyance. If Participant 4 was annoyed with her husband, she did not usually make sexual relationship with her husband and if she did, she could not reach an orgasm. Participant 10 has always been angry and annoyed with her opium addicted husband and did not make sexual relationship because of fear of their children’s imagination but when she made a relationship under duress, she pulled her own hair and hit her head and permanently told him “hurry up, end it…!” As such, she had never experienced an orgasm in her sexual life with her spouse. 

**Speaking near orgasm moment that distracts** **the mind**

Participant 1 mentioned talking during sex: “Talking and expressing romantic words in the beginning of a sexual relationship will make the partners arouse and improve the sexual relationship but near an orgasm moment, talking prevents from focusing on pleasure. It is better to use sign language or sounds”. 

**Contextual obstacle**

**Irregular sleeping hours**

Participant 2 said: "Taking care of children and breastfeeding are the causes of my fatigue and irregular sleep pattern that has affected the number of times we make a sexual relationship and its subsequent pleasure”. Participant 18 said: "One of my sons sleeps late, also my sleeping hours are not matched with those of my husband and consequently we can not make a relationship comfortabl.”

**Lack of privacy at home**

- Small house without a private bedroom

Due to economic problems, Fariba cannot afford a larger house with a private bedroom: "It's so hard, we can only have sex when the kids are not at home and when we have sex, fear of the kids’ arrival makes us have a quick and hurried sexual relationship. That’s why I have never enjoyed our relationship ".

- Infant sleeping in the parents' bedroom

Participant 5 said: “Our house has 2 bedrooms, but my baby sleep in our bedroom for breastfeeding, for this reason, I cannot have sex easily and I cannot reach the climax”.

- Dedicate the only bedroom of house to the children

Participant 11 says: "When we went to the houses of our relatives, my 5-year old son saw that his peers had separate bedrooms, so we gave the only bedroom of our house to our son because we were afraid that he will feel a sense of lack of the relatives’ kids. Now, we sleep in the living room at night, .... so our relationships are reduced in number with less pleasure”.

**Lack of peace at home**

This factor was an obstacle to orgasm for Participant 13: “In order to be satisfied, I should make a relationship at an appropriate time and place, when complete peace and safety governs our house, not in the bathroom or when our children are watching cartoons or we have guests in the house”.

**Table I:** Participant's socio-demographic characteristics

	**Age (Year)**	**Educational level**	**Occupation**	**Duration of marriage**	**Number. of children**
Participant 1	19	Theology student	Housewife	1	0
Participant 2	27	Bachelor’s degree	Housewife	7	2
Participant 3	28	High school Diploma	Nurse-Aid	2	0
Participant 4	28	High school Diploma	Housewife	5	0
Participant 5	28	Associate’s degree	Housewife	8	1
Participant 5	31	High school Diploma	Housewife	8	2
Participant 7	32	Master’s degree	Housewife	4	1
Participant 8	33	Bachelor’s degree	Housewife	12	2
Participant 9	34	PhD student	Teacher	13	2
Participant 10	36	Illiterate	Housewife	20	3
Participant 11	38	Bachelor’s degree	Teacher	7	2
Participant 12	38	Bachelor’s degree	Consultant	17	1
Participant 13	38	Bachelor’s degree	Employee	19	2
Participant 14	40	Master’s degree student	Consultant	18	2
Participant 15	41	Master’s degree	Employee	16	2
Participant 16	41	Master’s degree student	Housewife	21	2
Participant 17	43	Master’s degree	Teacher	19	0
Participant 18	43	Bachelor’s degree	Employee	15	2
Participant 19	44	Bachelor’s degree	Teacher	24	2
Participant 20	48	High school Diploma	Housewife	30	2

**Table II:** Codes, subcategories, and categories derived from the results of the present study

**Category**	**Subcategory**	**Code**
Multi dimensionality of women’s orgasm obstacles	Physical obstacles	Physical fatigue of the husband or wife Vaginal infection Insufficient vaginal lubrication
	Psychological obstacles	Lack of sexual Knowledge Shameof expressing her sexual demands Shameof doing some sexual behaviors Lack of concentration on sex Concern about the household and children problems Concern about children' s imagination about parents’ sex Wife’s concern about her husband’s satisfaction Fear of sexual intercourse Fear of pregnancy Anxiety of orgasm failure recurrence
	Relational obstacles	Husband’s hurry Having a dispute and annoyance with the spouse Speaking close to the orgasm moment that distracts the mind
	Contextual obstacles	Irregular sleeping hours Lack of privacy at home Small house without bedroom Infant sleeping in the parents' bedroom Dedicating the only bedroom of the house to the children Lack of peace at home

## Discussion

This study explores the obstacles impacting orgasm among Iranian women. Orgasm obstacles include a wide range of physical, psychological, relational and contextual factors.

**Physical obstacles to women's orgasm**

In this study physical fatigue of the husband or wife, vaginal infection and vaginal dryness were mentioned as physical obstacles. In this research, physical fatigue due to daily works was listed as woman’s orgasm obstacles. Another Iranian study also reported more fatigue in women with the orgasmic disorder (10). The related literature mainly refers to the impact of fatigue on sexual performance such as fatigue arising from chronic diseases (30) or babysitting (31). Anyhow, fatigue, whether as a result of chronic diseases or daily activities, has an obstructive effect on sexual performance and consequently orgasm. 

Like the present study, in other studies, women’s orgasm was obstructed by vaginal infections. In the study of Lopez on 399 women with vaginal infection, 21 percent reported orgasm failure (32).

Vaginal dryness may be caused by atrophic vaginitis due to hypostrogenemic conditions in menopause or after breastfeeding (33). Vaginitis atrophic, especially during its early incidence, can be treated (34). Despite the frequent recourse of women to healthcare specialists, women usually do not talk about a sexual issue unless they are asked (35). As a result, asking about women’s sexual status, providing knowledge and training for breastfeeding or menopausal women and treatment of atrophic vaginitis can be considered essential sexual interventions after delivery or near the menopause age. Insufficient vaginal lubrication in the participants of the present study had occurred mainly following postmenopausal or breastfeeding changes. Perhaps insufficient foreplay before the intercourse is the cause of vaginal dryness in some women, that should be taken into account in couple’s training (36). 

Drugs are biological factors that affect women’s orgasm. In the present study, participants did not use any drug that would affect their sexual function, but according to the literature, some medications can obstruct women’s orgasm and selective serotonin reuptake inhibitors (SSRIs) are the most important drugs that delay or obstruct female orgasm (37, 38). Although some medications have been studied for improvement of female orgasm, there is still controversy about their effectiveness and safety (39). A review of the literature from 1970 to 2014 indicated that 90 percent of the interventions for orgasmic disorder were non-pharmacological (40). In the last literature review, conducted by “up-to-date website”, medications had no place in the treatment of primary female orgasm disorder and orgasm disorder therapy mainly included education and psychosocial interventions; So gathering enough psychosexual history and knowledge about possible causes or contributing factors in the context of patients’ real life are essential (6).

**Psychological obstacles of woman’s orgasm**

Psychological obstacles in this study included lack of sexual knowledge, shame, lack of concentration on sex, fear of sexual intercourse, fear of pregnancy, and anxiety of orgasm failure recurrence. 

Lack of knowledge and embarrassment were the orgasmic obstacles in the present study. Similarly, in another study in Iran, lack of sexual knowledge and shyness were higher in the orgasmic disorder group (10). In another Iranian study, fear of sex and the need for more training and experience were reported among the newly married women (41). The reason for fear of sexual intercourse might be having a history of sexual trauma (rape and sexual harassment, female genital mutilation) or the cultural beliefs that would lead to having a feeling of shame and guilt for having sex (42). Fear of sexual intercourse in the present study was mainly due to lack of knowledge and cultural teachings. Some of these psychological obstacles that are rooted in cultural beliefs can be removed through increasing sexual education for couples.

Woman’s lack of concentration and inability to focus on the sexual relationship were the major psychological obstacle to orgasm in the present study. In another Iranian study that was carried out by Bokaie et al, intrusive thoughts caused problems in the sexual response cycle and orgasm (43). It appears that teaching relaxation and mindfulness techniques are effective in improving women’s orgasm in Iran.

In a study among medical students in Germany, contraception was an important factor affecting female sexual function index (44). In young Chinese women, the pressure for getting pregnant and not using contraceptive methods was associated with female sexual dysfunction (45). Similarly, in the present study, fear of unwanted pregnancy was one of the causes of not reaching an orgasm; so contraceptive methods consultation tailored to the age and lifestyle can be effective in improving women’s orgasm.

Increased anxiety is associated with an increased difficulty in reaching an orgasm and decreased anxiety is one of the important components of the treatment for the orgasmic disorder (46-47). In the present study, some participants (Participant 1 and Participant 4) reported anxiety for recurrence of not reaching orgasm at the beginning of the treatment that had been gradually resolved through focusing on positive thoughts.

An Indonesian researcher pointed out that some women, due to cultural teachings believed that they should be a sexual servant to their husband and should not care for and value their own pleasure. This ideology can be overcome by establishing an equal and symmetrical relationship between the husband and wife (48). In this study, only one of the participants was worried about her husband’s dissatisfaction during the early days of marriage that had been removed gradually following the promotion of couple’s intimacy. So training the couples about equal and intimate relationships can help overcome this problem. 

**Relational obstacles to women’s orgasm**

The method of interaction between the husband and wife would affect woman’s orgasm. In the present study, the husband’s hurry, dispute and annoyance with him and talking at the moments just before orgasm that distracted their focus were counted as relational obstacles. It seems that psychological and relational (dispute and annoyance with husband) factors had a more significant effect on orgasm than physical, physiological and reflexive factors and despite the performance of appropriate stimulation they have made reaching an orgasm difficult or sometimes even impossible. This finding is consistent with Basson’s model for female sexual response. From Basson’s point of view, women’s sexual response is more complicated than that of men, which is affected by various sexual and non-sexual grounds such as satisfaction with marital relationship, emotional intimacy and sexual arousal (49). Mary and Kelly mentioned some relational factors that would obstruct female orgasm, like blaming and less receptivity (13). Other researchers also have considered orgasm as a learnable experience and according to the culture and norms in Iranian society, couple’s therapy and sexual skills training, from among the existing therapies for female orgasmic disorders, were considered more appropriate treatments for orgasm disorders (40). 

**Contextual obstacles to women’s orgasm**

Contextual obstacles to women’s orgasm in the present study mainly were related to the lifestyle, including irregular sleeping hours, and lack of privacy and peace at home. Other studies also emphasized that in addition to physical factors, psychological, cultural and social factors, would influence female orgasm too (50-52). In a study in Singapore, in addition to physiological factors, sociocultural factors and lifestyle, were also effective in the sex life (53). The impact of lifestyle on sexual performance has been reported in the middle-aged women 53,54). Similarly, in the present study, the impact of lifestyle was greater in middle-aged women (Participant 13., Participant 20, Participant 10).

Concerning sleeping hours, other studies have reported that longer sleep duration was related to increased sexual desire during the next day. Sufficient sleep hours is so important in sexual desire and arousal that sleep disorder may be considered as a risk factor for sexual dysfunction in future (55). In the present study, complaints about irregular sleep hours were mainly reported by breastfeeding mothers. Helping these mothers with their motherhood duties, even if it only adds one extra hour to the mother's hours of sleep, can improve female sexual function.

**Notable findings in the cultural context of Iran**

Lack of sexual knowledge, tiredness, and bedroom-related problems could be referred to as some of the findings of the present study in the cultural context of Iran that worth thinking, planning and educating. Even though nowadays, women have higher educational degrees and access to internet and mass media, there are lots of couples who lack knowledge about how to make a sexual relationshipIt seems that sparse training on different internet websites and virtual groups and channels are not enough and the need for scientific policies and national planning on sexual health training is felt. In another Iranian study, Khalesi *et al* emphasized on sexual education and sexual health services at governmental centers (56). 

When the participants of the present study were asked “What occasions would make it more difficult for them to reach an orgasm?”, they considered tiredness as the first answer. Fatigue was obvious both in working women and housewives. Tiredness among working women is justifiable, but in the housewives, it seems odd at the first glance. However, the fact is that with respect to the shrinking of family dimensions and lack of presence of grandparents and other close relatives in today Iranian households, sharing of responsibilities among family members is less and all the housekeeping, cooking and nursing responsibilities are for the wife. 

As the father is at work from dawn to dusk and cannot help his wife, the housewife’s tiredness is not strange. Women expressed that when their mother, their sister, a babysitter, or a maid helped them with the housework and babysitting, they were less tired when entering the bedroom and were more eager to make a sexual relationship with their spouse and it was more probable for them to reach the climax. In this study, the husband’s tiredness had an obstructive effect on the woman’s orgasm too, probably because a tired man did not have the physical and psychological power for romantic foreplay to prepare his wife sexually. Accordingly, in couples counseling and education, the balance between daily work and night’s sleep and a disciplined lifestyle should be emphasized to promote the quality of sexual relationships and reach an orgasm. 

**Exclusive finding of this study**

The exclusive results of the present study which had no similarity in previous studies included the problems related to the bedroom and the inability to allocate a privacy of the parents and children. One of the requirements for having a good sex is to have privacy and a suitable bedroom. Some of the families, due to economic conditions, lived in a small house without any bedroom so that it was too difficult for them to make a sexual relationship. However, some participants had one or two bedrooms, but the couple failed to use them properly. As long as their children were infants and being breastfed, the parent’s concern with the baby’s conditions was a reason for their failure to use a private bedroom. When the children grew older, fear of children’s imagination of sex between their parents was an obstacle. A separate sleeping place for children must be stressed in couples’ training. Moreover, this culture should be promoted that if there is only one bedroom in the house, it should be dedicated to the parents. The holy Quran also emphasizes on a specific privacy for the parents and the necessity of asking for permission by the children to enter their parents’ bedroom (Surah Noor, verse 59). 

Likewise, parents should be taught that they are responsible for making a relationship out of their children’s sight, although the children’s imagination is beyond parent’s control. In those families with mature and young children, a private bedroom with a closed door for parents may make children believe that their parents have an intimate and warm relationship. This issue can leave positive impacts on the children’s future marital life. 

**Limitations**

As talking about sexual issues and particularly orgasm was considered a taboo in Iranian society, it was a time-consuming task to attract the participants’ trust and assure them about the confidentiality of their information.

## Conclusion

The current paper explained different physical, psychological, relational and contextual aspects affecting female orgasm from the standpoints of Iranian women. Among physical obstacles, wife’s or husband’s fatigue was impressive. Lack of sexual knowledge, shame, and lack of concentration on sex due to household and children's problems were the major psychological obstacles to a woman’s orgasm. Husband’s hurry was mentioned as one the relational obstacles. Lack of privacy and inability to separate children’s bedroom from their parents were the main contextual obstacles to women’s orgasm. Many of these obstacles can be adjusted by training and counseling of couples before and during the marriage, practicing in mindfulness techniques and making some changes in the lifestyle of the couples. Concerning the major contribution of non-physical orgasm obstacles and the greater success of non-pharmacological treatments compared to pharmacological therapies, further studies related to psychological, relational and contextual obstacles based on the culture of each society seems necessary in order to prevent or treat female orgasm disorders.

## Note

***This article extracted from a Ph.D. thesis*** ***(Maryam Nekoolaltak)***

## References

[1] Balon R, Segraves RT (2005). Handbook of sexual dysfunction.

[2] Rossi AS Eros and Caritas: A biopsychosocial approach to human sexuality and reproduction. Sexuality across the life course.

[3] Emhardt E, Siegel J, Hoffman L (2016). Anatomic variation and orgasm: Could variations in anatomy explain differences in orgasmic success?. Clin Anat.

[4] Gravina GL, Brandetti F, Martini P, Carosa E, Di Stasi SM, Morano S (2008). Measurement of the thickness of the urethrovaginal space in women with or without vaginal orgasm. J Sex Med.

[5] Wallen K, Lloyd EA (2011). Female sexual arousal: Genital anatomy and orgasm in intercourse. Horm Behav.

[6] Treatment of female orgasmic disorder.

[7] Harris JM, Cherkas LF, Kato BS, Heiman JR, Spector TD (2008). Normal variations in personality are associated with coital orgasmic infrequency in heterosexual women: A population‐based study. J Sex Med.

[8] Kelly MP, Strassberg DS, Kircher JR (1990). Attitudinal and experiential correlates of anorgasmia. Arch Sex Behav.

[9] Dunn KM, Croft PR, Hackett GI (1999). Association of sexual problems with social, psychological, and physical problems in men and women: a cross sectional population survey. J Epidemiol Community Health.

[10] Najafabady MT, Salmani Z, Abedi P (2011). Prevalence and related factors for anorgasmia among reproductive aged women in Hesarak, Iran. Clinics.

[11] Kelly MP, Strassberg DS, Turner CM (2004). Communication and associated relationship issues in female anorgasmia. J Sex Marital Ther.

[12] Nekoolaltak M, Keshavarz Z, Simbar M, Nazari AM (2016). Sexual talk with the spouse: Sarcastic or Soothing?. Int J Hum Cultur Stud.

[13] Kelly MP, Strassberg DS, Turner CM (2006). Behavioral assessment of couples' communication in female orgasmic disorder. J Sex Marital Ther.

[14] Frederick DA, Lever J, Gillespie BJ, Garcia JR (2017). What Keeps Passion Alive? Sexual Satisfaction Is Associated With Sexual Communication, Mood Setting, Sexual Variety, Oral Sex, Orgasm, and Sex Frequency in a National US Study. J Sex Res.

[15] Frith H (2015). Sexercising to orgasm: Embodied pedagogy and sexual labour in women’s magazines. Sexualities.

[16] Frith H (2013). Labouring on orgasms: embodiment, efficiency, entitlement and obligations in heterosex. Cult Health Sex.

[17] Klapilová K, Brody S, Krejčová L, Husárová B, Binter J (2015). Sexual satisfaction, sexual compatibility, and relationship adjustment in couples: the role of sexual behaviors, orgasm, and men's discernment of women's intercourse orgasm. J Sex Med.

[18] Hurlbert DF, White LC, Powell RD, Apt C (1993). Orgasm consistency training in the treatment of women reporting hypoactive sexual desire: An outcome comparison of women-only groups and couples-only groups. J Behav Ther Exp Psychiatr.

[19] Mark KP, Milhausen RR, Maitland SB (2013). The impact of sexual compatibility on sexual and relationship satisfaction in a sample of young adult heterosexual couples. Sex Relat Ther.

[20] Rahmani A, Merghati Khoei E, Alahgholi L (2009). Sexual satisfaction and its relation to marital happiness in Iranians. Iran J Public Health.

[21] Merghati-Khoei E, Zargham-Boroujeni A, Salehi M, Killeen TK, Momeni G, Pasha Y (2015). Saturated love leading to sexual pleasure: Iranian women's narratives. Caspian J Appli Sci Res.

[22] Gałecki P, Depko A, Jȩejewska D, Talarowska M (2012). Human orgasm from the physiological perspective - Part I. Pol Merkur Lekarski.

[23] Parker R (2009). Sexuality, culture and society: shifting paradigms in sexuality research. Cult Health Sex.

[24] Guarte JM, Barrios EB (2006). Estimation under purposive sampling. Commun Stat Simulat Comput.

[25] Miles J, Gilbert P (2005). A handbook of research methods for clinical and health psychology.

[26] Wiles R, Crow G, Heath S, Charles V (2006). Anonymity and confidentiality.

[27] Hsieh HF, Shannon SE (2005). Three approaches to qualitative content analysis. Qual Health Res.

[28] Guba EG (1981). Criteria for assessing the trustworthiness of naturalistic inquiries. ECTJ.

[29] Guba EG, Lincoln YS (1989). Fourth generation evaluation.

[30] Blazquez A, Ruiz E, Aliste L, García-Quintana A, Alegre J (2015). The effect of fatigue and fibromyalgia on sexual dysfunction in women with chronic fatigue syndrome. J Sex Marital Ther.

[31] Yeniel AO, Petri E (2014). Pregnancy, childbirth, and sexual function: perceptions and facts. Int Urogynecol J.

[32] López-Olmos J (2012). Infecciones vaginales y lesiones celulares cervicales (III). Características de la sexualidad. Clin Invest Ginecol Obstet.

[33] Palmer AR, Likis FE (2003). Lactational atrophic vaginitis. J Midwifery Womens Health.

[34] Domoney C (2014). Treatment of vaginal atrophy. Womens Health (Lond).

[35] McDonald E, Woolhouse H, Brown SJ (2015). Consultation about Sexual Health Issues in the Year after Childbirth: A Cohort Study. Birth.

[36] Shaeer O, Shaeer K, Shaeer E (2012). The Global Online Sexuality Survey (GOSS): Female sexual dysfunction among Internet users in the reproductive age group in the Middle East. J Sex Med.

[37] Ben-Sheetrit J, Aizenberg D, Csoka AB, Weizman A, Hermesh H (2015). Post-SSRI sexual dysfunction: Clinical characterization and preliminary assessment of contributory factors and Dose-Response relationship. J Clin Psychopharmacol.

[38] Waldinger MD (2015). Psychiatric disorders and sexual dysfunction. Handb Clin Neurol.

[39] Nappi RE, Cucinella L (2015). Advances in pharmacotherapy for treating female sexual dysfunction. ExpOpin Pharmacother.

[40] Salmani Z, Zargham-Boroujeni A, Salehi M, Killeen TK, Merghati-Khoei E (2015). The existing therapeutic interventions for orgasmic disorders: recommendations for culturally competent services, narrative review. Iran J Reprod Med.

[41] Ibrahimipure H, Jalambadani Z, Najjar AV, Dehnavieh R (2012). The first experience of intercourse in married women of Sabzevar city: a phenomenological study. Health Med.

[42] El-Hadidy MA, Eissa A, Zayed A (2016). Female circumcision as a cause of genophobia. Middle East Curr Psychiatr.

[43] Bokaie M, Simbar M, Yassini Ardekani SM (2015). Sexual behavior of infertile women: A qualitative study. Iran J Reprod Med.

[44] Wallwiener CW, Wallwiener LM, Seeger H, Muck AO, Bitzer J, Wallwiener M (2010). Prevalence of sexual dysfunction and impact of contraception in female German medical students. J Sex Med.

[45] Du J, Ruan X, Gu M, Bitzer J, Mueck AO (2016). Prevalence of and risk factors for sexual dysfunction in young Chinese women according to the Female Sexual Function Index: an internet-based survey. Eur J Contracept Reprod Health Care.

[46] De Lucena BB, Abdo CHN (2014). Personal factors that contribute to or impair women's ability to achieve orgasm. Int J Impot Res.

[47] Meston CM, Hull E, Levin RJ, Sipski M (2004). Disorders of orgasm in women. J Sex Med.

[48] Nurmila N (2013). Indonesian Muslims’ Discourse of Husband-Wife Relationship. Al-Jami'ah: J Islam Stud.

[49] Basson R (2000). The female sexual response: A different model. J Sex Marital Ther.

[50] Faubion SS, Rullo JE (2015). Sexual Dysfunction in Women: A Practical Approach. Am Fam Physician.

[51] Thomas HN, Thurston RC (2016). A biopsychosocial approach to women’s sexual function and dysfunction at midlife: A narrative review. Maturitas.

[52] Rao TS, Nagaraj AK (2015). Female sexuality. Indian J Psychiatry.

[53] Goh VH, Tain CF, Tong YY, Mok PP, Ng SC (2004). Sex and aging in the city: Singapore. Aging Male.

[54] Nappi RE, Albani F, Valentino V, Polatti F, Chiovato L, Genazzani AR (2007). Aging and sexuality in women. Minerva Ginecol.

[55] Kalmbach DA, Arnedt JT, Pillai V, Ciesla JA (2015). The impact of sleep on female sexual response and behavior: A pilot study. J Sex Med.

[56] Khalesi ZB, Simbar M, Azin SA, Zayeri F (2016). Public sexual health promotion interventions and strategies: A qualitative study. Electron Physician.

